# Facilitating exit from encampments: combining low-barrier transitional housing with stabilizing treatment for substance related problems

**DOI:** 10.1186/s13722-023-00420-y

**Published:** 2023-10-26

**Authors:** Miriam Komaromy, Andrea Stone, Alicia Peterson, Jacqueline Gott, Rob Koenig, Jessica L. Taylor

**Affiliations:** 1 Boston Medical Center, Boston, MA USA; 2 Grayken Center for Addiction, Boston Medical Center, Boston, MA USA; 3 Boston University, Boston University Chobanian & Avedisian School of Medicine, Boston, MA USA

**Keywords:** Transitional housing, Substance use disorder treatment, Homelessness, Tent encampments, Harm reduction

## Abstract

**Background:**

Tent encampments in the neighborhood surrounding Boston Medical Center (BMC) grew to include 336 individuals at points between 2019 and 21, prompting public health concerns. BMC, the City of Boston, and Commonwealth of Massachusetts partnered in 2/2022 to offer low-barrier transitional housing to encampment residents and provide co-located clinical stabilization services for community members with substance use disorders (SUDs) experiencing homelessness.

**Methods:**

To meet the needs of some of the people who had been living in encampments, BMC established in a former hotel: 60 beds of transitional housing, not contingent upon sobriety; and a low-barrier SUD-focused clinic for both housing residents and community members, offering walk-in urgent care, SUD medications, and infection screening/prevention; and a 24/7 short-stay stabilization unit to manage over-intoxication, withdrawal, and complications of substance use (e.g., abscesses, HIV risk, psychosis). A secure medication-dispensing cabinet allows methadone administration for withdrawal management. Housing program key metrics include retention in housing, transition to permanent housing, and engagement in SUD treatment and case management. Clinical program key metrics include patient volume, and rates of initiation of medication for opioid use disorder.

**Results:**

Housing: Between 2/1/22–1/31/2023, 100 people entered the low-barrier transitional housing (new residents admitted as people transitioned out); 50 former encampment residents and 50 unhoused people referred by Boston Public Health Commission. Twenty-five residents transferred to permanent housing, eight administratively discharged, four incarcerated, and four died (two overdoses, two other substance-related). The remaining 59 residents remain housed; none voluntarily returned to homelessness. One hundred residents (100%) engaged with case management, and 49 engaged with SUD treatment. Clinical: In the first 12 months, 1722 patients (drawn from both the housing program and community) had 7468 clinical visits. The most common SUDs were opioid (84%), cocaine (54%) and alcohol (47%) and 61% of patients had a co-occurring mental health diagnosis in the preceding 24-months. 566 (33%) patients were started on methadone and accepted at an Opioid Treatment Program (OTP).

**Conclusions:**

During the 1st year of operation, low-barrier transitional housing plus clinical stabilization care was a feasible and acceptable model for former encampment residents, 49% of whom engaged with SUD treatment, and 25% of whom transitioned to permanent housing.

## Background

People experiencing homelessness often have severe untreated substance use disorders (SUDs), and experience SUD-related barriers to, and discrimination in, obtaining temporary shelter, post-acute care [1], and housing [2–5]. In Boston, as in most communities, public shelters do not permit substance use in the shelter, and those who leave the shelter overnight are not permitted to return [6]. Fentanyl, which is ubiquitous in Boston and requires frequent use to avoid withdrawal symptoms, therefore renders the shelter system inaccessible to many people with SUD [7, 8]. People with SUD are also effectively excluded from many subsidized housing programs; for example, the Boston Housing Authority operates more than 4500 units for low-income Bostonians but, per federal regulation, requires that tenants do not use substances [9–12]. This policy can be associated with residents misrepresenting SUD status, which in turn results in increased risk of overdose death [13].

People experiencing homelessness who are not able to access shelter or housing often band together for safety and create tent encampments. Although Boston has not historically had year-round encampments, fixed encampments emerged intermittently in the neighborhood adjacent to Massachusetts Avenue and Melnea Cass Boulevard (termed “Mass and Cass”) in the years leading up to the COVID-19 pandemic, driven by fentanyl-related barriers to shelter utilization and the 2014 closure of a large, nearby addiction treatment program and shelter. Attempts by earlier Boston City administrations to clear the encampments [14] with strategies including demolition, increased policing, and incarceration were associated with decreases in both SUD treatment utilization and harm reduction service delivery [15, 16]. These approaches did not lead to sustained disappearance of encampments.

The COVID-19 pandemic created additional barriers to shelter utilization, including concerns about transmission in congregate settings, and by Fall of 2021, tent encampments in the “Mass and Cass” neighborhood, where visible substance use and homelessness are most concentrated, had grown to include 336 individuals. Challenges included lack of physical safety, fires in tents [17], rodent infestation linked to a case of human leptospirosis [18], frequent opioid overdose, ongoing high rates of HIV transmission [19], and injection drug use that was frequently visible to the public. This situation prompted humanitarian, public health, and community concerns.

The Mayoral administration elected in November of 2021 committed to housing encampment residents [20], and the City of Boston contracted with several organizations to provide transitional housing. Though this paper refers to “transitional housing”, the housing model was referred to by the City of Boston as “crisis housing” and has elements of both emergency shelter and transitional housing. Boston Medical Center (BMC), the City’s largest and oldest safety-net hospital, located blocks away from the encampments, leased a vacant hotel and partnered with the City to operate 60 beds of transitional housing beginning in February 2022. Unlike other local shelter and housing models, transitional housing residency was not contingent on residents’ substance use or treatment engagement.

**Fig. 1:**
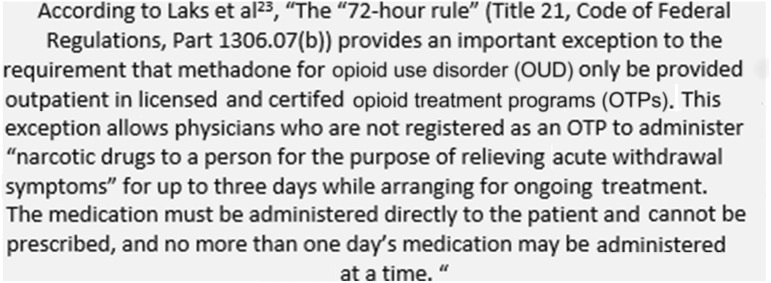
72-h rule for administration of methadone for emergency opioid withdrawal management

The Commonwealth of Massachusetts also funded BMC to provide co-located acute SUD stabilization services for people experiencing homelessness, including a low-barrier SUD-focused clinic offering walk-in urgent care, medications for SUD, and infection screening/prevention; and a 24/7 short-stay stabilization unit to manage over-intoxication (including both over-sedation and over-stimulation/over-amping [21]), withdrawal, and other complications of substance use (e.g., abscesses, HIV risk, psychosis). A secure dispensing cabinet allowed methadone administration for withdrawal management under provisions of the federal 72-h rule [22, 23] (Fig. 1). The clinical services were available to housing residents and also other community members experiencing homelessness.

The goal of this paper is to describe the model of co-located low-threshold transitional housing and SUD clinical services for people exiting tent encampments. We describe initial housing metrics, including transitional housing retention, transition to permanent housing, and engagement in SUD treatment and case management; and initial clinical services outcomes, including patient volume, rates of initiation of methadone for opioid use disorder (OUD), and care utilization.

*Note that the authors played clinical and administrative roles in the design, launch, and operation of the transitional housing and clinical programs, and that these relationships pose potential conflicts of interest. MK and JT serve as Co-Medical Directors for the clinical program and AP serves as Operations Director. All three were involved in all phases of the transitional housing and clinical programs. JG serves as Nurse Manager for the clinical program. RK is Executive Director of Strategic Programs for BMC and oversaw planning, implementation, and operations for the transitional housing and clinical programs; he also led efforts to secure stable funding for the programs. All authors are employed by Boston University or its primary teaching hospital, Boston Medical Center, and the housing and clinical services operate as a program of Boston Medical Center. Funding for the transitional housing programs was provided by the Boston Public Health Commission, and the City of Boston. Funding for the clinical services was provided from the Massachusetts Commonwealth Bureau of Substance Addiction Services (BSAS), City of Boston,, Boston Public Health Commission, and by BMC. Clinical program costs were nominally offset by billing Massachusetts Medicaid (MassHealth). No external clinical or political entity was given the opportunity to preview or edit this manuscript prior to submission.*

## Methods

### Design

Co-located low-threshold transitional housing and clinical services were implemented urgently to address worsening conditions in Boston tent encampments in February 2022. We describe the model, key transitional housing metrics, and preliminary clinical service outcomes. This work was approved by the Boston University Medical Campus Institutional Review Board (protocol H-43035).

### Setting

BMC established the following services in a former hotel known as the “Roundhouse (RH)” in February 2022:

1. Low-threshold, transitional housing

The RH offers sixty beds of transitional housing for tent encampment residents, with transitional housing eligibility not contingent upon abstinence from substances or SUD treatment engagement. The Boston Public Health Commission (BPHC) refers residents from encampments and manages a waiting list of former encampment residents and other people experiencing homelessness. Rooms are single or double occupancy with private bathrooms and have locking doors. The RH transitional housing program accepts individuals and couples, who have the option of being housed together or separately. Meals are delivered from City Fresh [24] and are available for pick up from the lobby three times daily.

RH transitional housing is staffed by case managers and harm reduction specialists. Due to the ongoing risk of overdose, staff provide around-the-clock safety checks of residents while they are in their rooms in order to help prevent overdose deaths. Harm reduction specialists also distribute harm reduction equipment (e.g., safer injection kits), link residents to community resources, and lead meetings and programming for residents. Case managers meet with residents individually and support connection to stabilization services and navigation to secure permanent supportive housing, based on need. The case manager-to-resident ratio is 1:15, with regular meetings scheduled between residents and their respective case managers on a weekly basis or as required, depending on the residents’ needs. Case managers provide assistance with preparing for, and transitioning to, permanent supportive housing, including obtaining identification documents, clearing warrants, and assisting residents to engage with clinical services in order to stabilize medical, mental health, and SUD issues. They also help enroll clients in benefits programs, provide conflict resolution, assist with family reunification, and provide emotional support.

Security guards are on site 24/7 and perform metal-detector searches for weapons when people enter the facility, but they do not search resident rooms or confiscate harm reduction equipment. Residents are provided with private “personal property lockers” external to the building, which are not searched. Residents are informed that substance use is prohibited in the building; if substances are observed (e.g., at the time of building entry), residents are reminded of facility policies and are guided towards using their lockers to store anything that they cannot bring inside of the building.

Roundhouse housing residents are also supported by other case management and clinical agencies. They are free to engage with any outside service agency, and in addition, several agencies provide personnel who come on-site to provide support. This includes Boston Healthcare for the Homeless Programs, which provides a half day per week of “home visits” to residents in their Roundhouse rooms, with a goal of enrolling residents in primary care or providing primary care services to residents who are not willing or able to attend clinical office visits. A MA Department of Mental Health (DMH) case manager is on site one half day per week, with a primary goal of enrolling residents who are DMH service-eligible into their programs. See Table 1 for a list of example external agencies that collaborate on providing clinical and case management stabilization services for people who are experiencing homelessness in the Mass and Cass area.

- 2. Clinical services (Table 2)

**Table 1:** Agencies and organizations that collaborate to provide services to people living unhoused in the Mass and Cass neighborhood of Boston

Services	Partner organization(s)
Housing navigation	▪ Eliot Community Human Services ▪ HomeStart (rapid re-housing agency) ▪ Boston Housing Authority ▪ Boston Medical Center
Harm reduction	▪ AHOPE: Access, Harm Reduction, Overdose Prevention and Education, a harm reduction and needle exchange site of BPHC ▪ SPOT: Supportive Place for Observation and Treatment, a daytime sedation monitoring program of Boston Healthcare for the Homeless ▪ Project TRUST: Harm reduction and street outreach program of Boston Medical Center ▪ Engagement Center: low-threshold drop-in program of the Boston Public Health Commission
Legal	▪ “Services over Sentences” program of North Suffolk Mental Health Association, supporting entry into treatment of SUD with subsequent dismissal or reduction of legal charges ▪ Boston Police Department ▪ Medical Legal Partnership Boston (MLPB)
Medical care	▪ Boston Health Care for the Homeless Program ▪ MA Department of Mental Health ▪ Boston Medical Center ▪ Boston Emergency Medical Services (EMS) ▪ Opioid Treatment Programs ▪ Health Care Resource Centers ▪ Comprehensive Treatment Centers ▪ Addiction Treatment Center of New England ▪ Bay Cove Human Services
Government	▪ City of Boston ▪ Boston Housing Authority ▪ Mayor’s Office of Housing ▪ Coordinated Response Team (CRT) ▪ Commonwealth of Massachusetts
Transitional/emergency housing coordination	▪ Pine Street Inn ▪ Victory Programs ▪ Eliot Community Human Services ▪ Boston Public Health Commission
Employment	▪ New Market Business Association

**Table 2:** Services offered in the Roundhouse clinical units

Walk-in urgent care clinic	24/7 Short-stay stabilization unit
Medications for SUDs • Buprenorphine, sublingual or subcutaneous (long-acting) • Naltrexone • Acamprosate • Others	**Same services as provided in urgent care, plus:**
Outpatient withdrawal management •Opioid withdrawal: 72-h rule methadone with OTP linkage • Opioid withdrawal:Buprenorphine • Alcohol withdrawal: Benzodiazepines	Management of over-intoxication • Sedation • Over-stimulation • Over-amping
Skin and soft tissue infection care • Abscess • Cellulitis • Wound care	Management of mild-moderate acute withdrawal
Infection Screening, Treatment, Prevention • HIV testing (rapid and phlebotomy) • PEP, PrEP • HCV testing • Vaccines • STI testing and treatment	Behavioral health assessments and triage • Onsite emergency mental health evaluations for voluntary admission to Crisis Stabilization Unit
Reproductive Health • Pregnancy testing • Contraception • Condoms	Post-overdose observation • Offered to patients as an alternative when refusing ED transfer post overdose • Medications to assist with withdrawal symptoms following naloxone administration in the community or onsite
Harm-reduction supplies and education, including naloxone	
Case management for treatment referral	
Connection to recovery coach services	

RH clinical services are located on the basement level of the facility and serve both housing residents and other people experiencing homelessness and SUD, who access services primarily on a walk-in basis. Patients are triaged to one of two options:

- Low-barrier SUD Walk-In Urgent Care Clinic

**Fig. 2:**
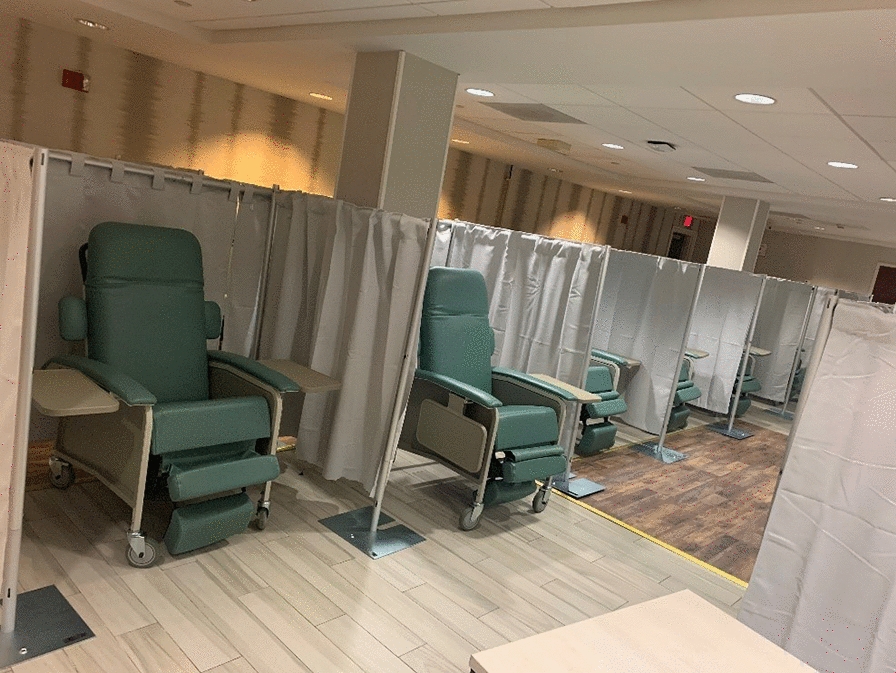
24/7 short-stay stabilization unit in converted hotel dining room, showing fully reclinable chairs for patient care

This outpatient clinic provides medications for SUD, infection treatment/screening/prevention services, contraception, and harm reduction education and supplies.

b. 24/7 Short-Stay Stabilization Unit (Fig. 2)

This “bedded outpatient” unit manages over-intoxication (both over-sedation and over-stimulation/over-amping), withdrawal, and complications of substance use (e.g., abscesses, HIV risk, psychosis). Patients are assigned to a recliner chair and may receive care for up to 24 h per treatment episode.

The clinical programs are staffed 24/7 with a nurse practitioner, two registered nurses, a registration clerk, and a harm reduction specialist. A secure medication dispensing cabinet allows for rapid administration of medications ordered through the electronic medical record, including methadone for opioid withdrawal management under the provisions of the 72-h rule [23] A case manager offers referral to ongoing treatment 7 days per week.

When patients present to one of the clinical programs with opioid withdrawal they often receive methadone treatment for their symptoms. Initiation of methadone treatment reflects emergency treatment of opioid withdrawal under the federal “72 h rule,” (see Fig. 1). Patients are eligible to return to the Roundhouse for additional methadone treatment of withdrawal symptoms for up to 72 h while being referred to ongoing care, most commonly at an OTP for long-term methadone treatment. Linkage refers to referral to an OTP that agrees to accept them for next day dosing and enrollment for ongoing care. Other patients requested referral to an inpatient medically managed withdrawal program (“detox”) or were referred for inpatient care of an acute medical condition.

### Data sources

Housing data were abstracted from data collection logs that were maintained prospectively by the case management team using a standardized data collection tool. Case managers updated the file after client contacts, which were scheduled to occur at least once weekly, and more frequently based on client need.

Clinical program and patient demographic data were abstracted from a standardized enterprise clinical data warehouse which is derived from the electronic health record. Monthly queries assessing medication orders, clinical encounter data and encounter diagnoses using BMC’s SUD and behavioral health ICD-10 code-sets (2-year diagnosis history) generated outcomes reported in this study. Methadone order data were obtained directly from Medication Administration Reports from the electronic health record, Epic (Epic Healthcare Systems, Verona, WI), and OTP referral and acceptance data were collected in flat files maintained by RH nursing staff, which were quality checked with chart review by program medical directors.

### Outcomes

Housing outcomes are reported for residents who entered RH housing during the 12-month period February 1, 2022–January 31 2023. Housing program key performance indicators include retention in transitional housing, transition to permanent housing, engagement in SUD treatment, and engagement in case management. Housing program residents were considered ‘retained’ if they resided in the RH for at least 3 months or transitioned to permanent supportive housing or a residential SUD treatment program. When calculating housing retention rates, residents who were incarcerated (n = 4) were excluded from the measure, as were those admitted to the RH less than 3 months before the end of the study period (n = 6).

Clinical program outcomes are reported for all patients who had an encounter at the RH clinical programs between February 1, 2022–January 31, 2023. In addition, separate clinical outcomes are reported for the subset of RH residents who also utilized RH clinical services.

Clinical program key performance indicators include visit volume and initiation on methadone for opioid use disorder. Due to historical data capture of Hispanic ethnicity as a race in our system, patients were considered Hispanic if they had Hispanic as a documented race or ethnicity. Race is presented as non-Hispanic White, non-Hispanic Black, Hispanic, and other race.

### Statistical methods

Descriptive statistics were used to summarize key outcomes. Not all residents will have had equal time to receive case management services, such as referral to permanent supportive housing, so summary statistics are provided for length of stay of housing program residents.

## Results

### Housing

In the first 12 months of operations, the RH admitted a total of 100 unsheltered people into the 60 beds of transitional housing, with turnover of 40 beds in the first 12 months. The individuals admitted into transitional housing included 50 encampment residents and 50 additional people experiencing homelessness.

The mean length of stay during the study period for RH residents (N = 100) was 247 days (range: 1–365 days, IQR: 252 days). Twenty-five residents (25%) were transferred to permanent supportive housing or other long-term housing placement, eight residents were administratively discharged due to violently disruptive behavior, four were incarcerated (one of whom returned to the RH after 5 months of incarceration), and four died. Presumed causes of death were two overdoses and two deaths related to chronic, substance-related illness.

Retention in transitional housing: Ten residents were not eligible to be evaluated for 3-month retention due to entering RH housing less than 3-months before the end of the study period (n = 6) and/or having their residence terminated by incarceration (n = 4). Among 90 residents eligible to be evaluated for the housing 3-month retention metric, 85 (94%) were retained in residence or transitioned to permanent supportive housing or other residential SUD treatment.

Five housing Case Managers completed 5140 visits (mean 1.4 visits/week per resident). All residents received case management services except one patient who died prior to initiating services. Case managers referred residents to a crisis stabilization unit, outpatient mental health care, recovery coaching, outpatient SUD treatment, harm reduction resources, residential rehabilitation services (withdrawal management, clinical stabilization services (CSS), transitional support services (TSS)), medication for addiction treatment (MAT) services, or the RH walk-in Urgent Care clinic.

### Clinical

**Fig. 3:**
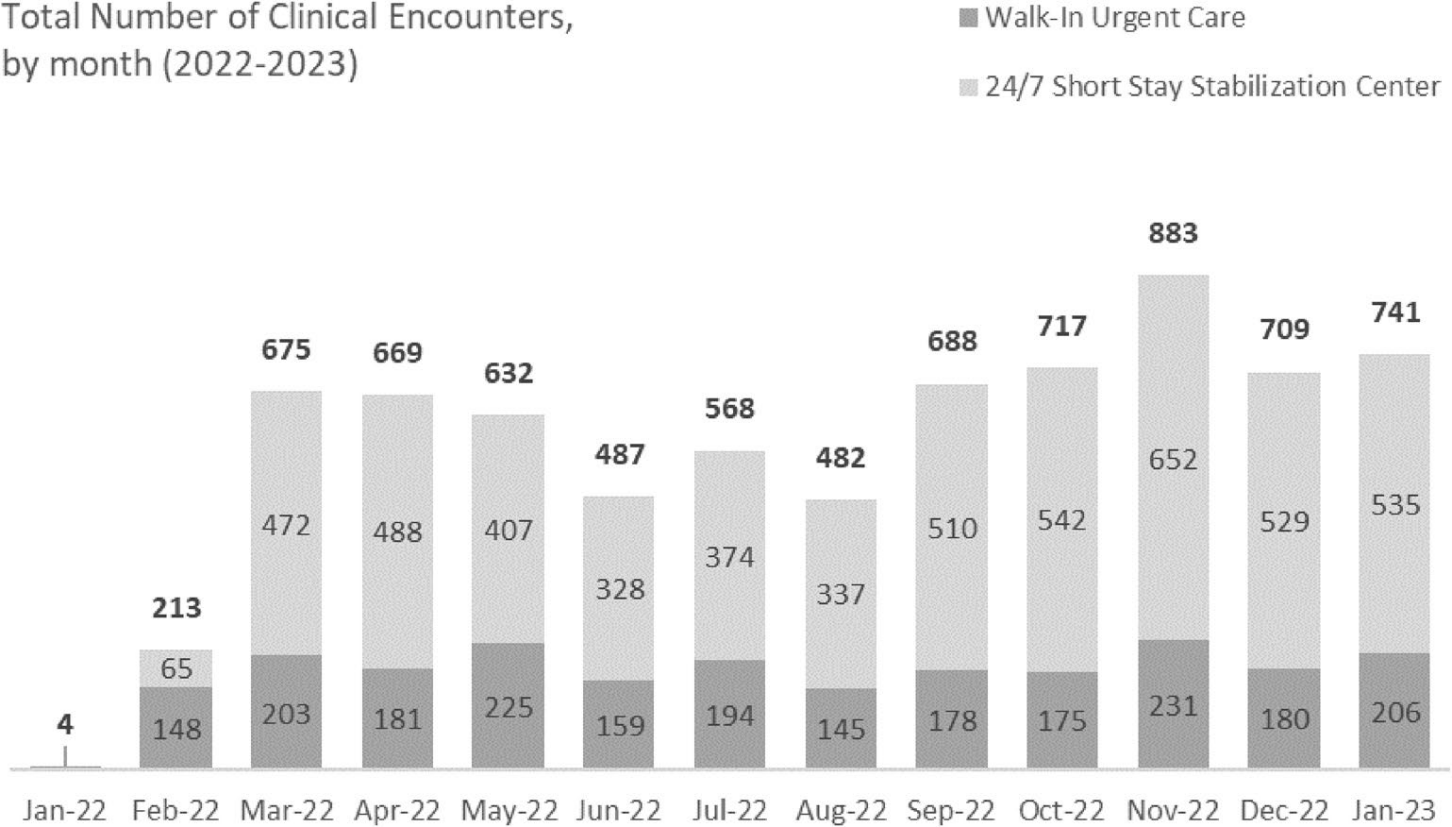
Volume of clinical services in the Roundhouse by month

In the first 12 months of operation, 1722 patients had 7468 visits (Fig. 3). The average length of stay for patients who received care in the 24/7 stabilization unit was 11.5 h. Patient demographics and utilization of clinical services by housing residents and overall are summarized in Table 3.

**Table 3:** Demographics of patients seen in Roundhouse clinical services, February 1, 2022– January 31, 2023, overall and housing resident subset

	Patients seen in roundhouse clinical programs	
	All	Housing residents only
Visit volume		
Unique patients	1722	59 ^
Total encounters	7468	425
Visits/Patient, mean (range, SD)	4.3 (1–99, 7.5)	7.2 (1–53, 7.4)*
Average length of stay in hours (range, SD)	11.5 (0–40, 5.7)	5.0 (0–38, 7.4)
Demographics		
Female, n (%)	467 (27.1%)	23 (39%)
Mean age in years (range, SD)	42.5 (19–79, 10.6)	40.7 (25–64, 9.2)
Race/Ethnicity, n (%)		
Non-hispanic white	1039 (60.3%)	28 (47.5%)
Non-hispanic black	369 (21.4%)	15 (25.4%)
Hispanic	211 (12.3%)	10 (16.9%)
Other race	102 (5.9%)	6 (10.2%)
Condition prevalence		
Opioid use disorder ^^^^	1441 (83.7%)	55 (93.2%)
Cocaine use disorder ^^^^	928 (53.9%)	43 (72.9%)
Alcohol use disorder ^^^^	808 (46.9%)	25 (42.4%)
MH Diagnosis (non-SUD dx)	1047 (60.8%)	38 (64.4%)
Service delivery		
Episodes of emergency methadone treatment for opioid withdrawal (72 h rule)	704	29
Episodes resulting in patients accepted to OTP after ≤ 72 h methadone induction ^^^^^	566	26

*MH* mental health, *SUD* substance use disorder, *OTP* opioid treatment program, *ED* Emergency Department

^ During the initial 12 months of program operation, a total of 100 people lived in the 60 available beds at the Roundhouse (n > 60 because of turn-over among residents). During this time 59 of the residents utilized the Roundhouse clinical services at least one time. These are the 59 people reflected in this table

^*^Roundhouse residents could utilize clinical services prior to, during, or after their time in residence, thus denominator of observation time is the same for all patients and equal to the study period (2/1/22–1/31/23)

^^SUD Diagnoses are assessed via a 2-year history of billed diagnoses in the electronic health record

^^^Metric reflects separate episodes of use of methadone to treat opioid withdrawal for up to 72 h. This does not represent all patients who were treated for opioid withdrawal in the Roundhouse, but only those who received methadone treatment

**Fig. 4:**
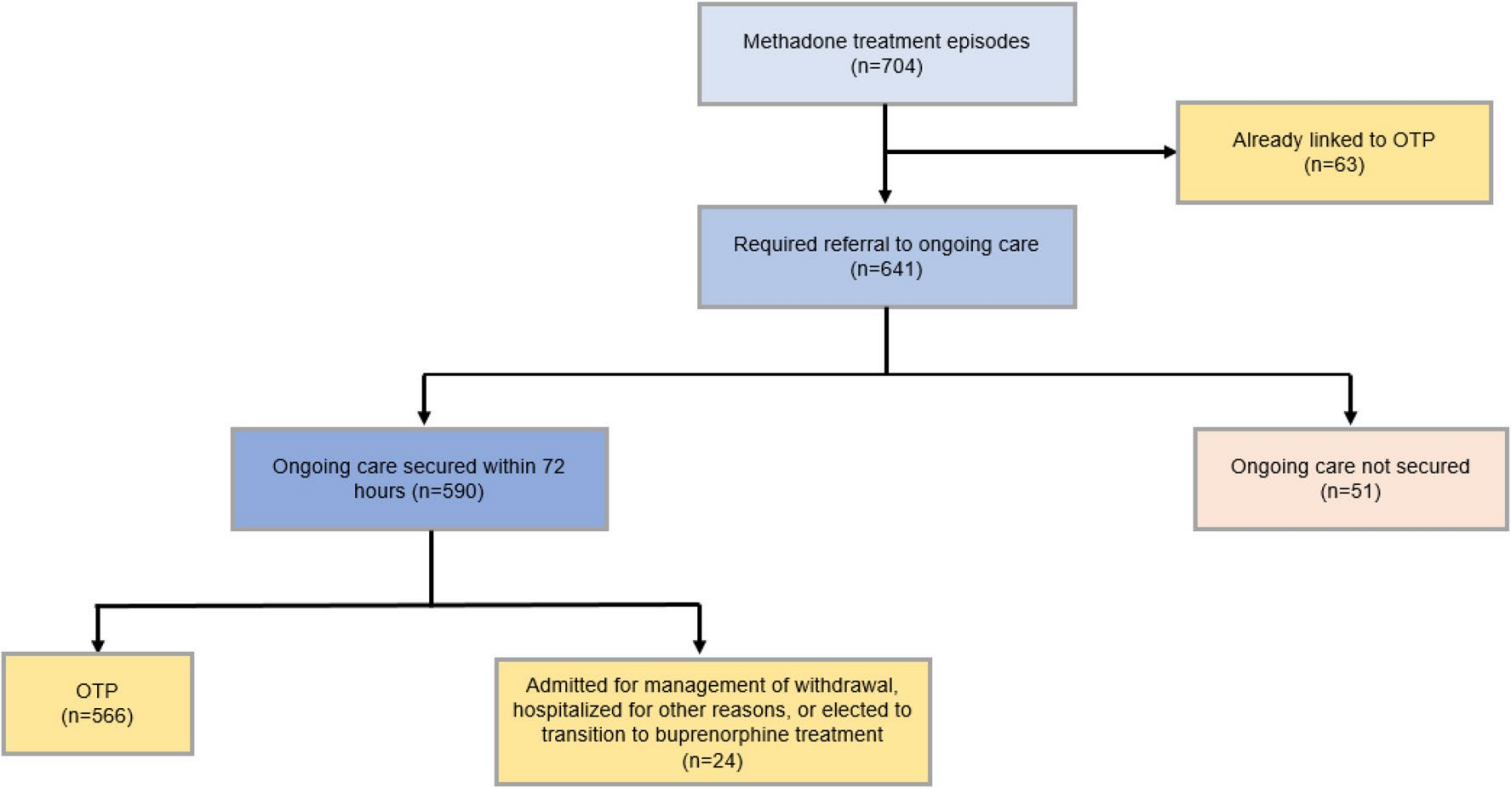
Plans for Ongoing Care for Methadone Treatment Episodes in Roundhouse clinical programs, Boston, MA: February 1, 2022–January 31, 2023

The most common SUD diagnoses were opioid (84%), cocaine (54%) and alcohol (47%). Many patients with opioid use disorder presented with opioid withdrawal, which was managed with a variety of medications. 554 patients who were seen in one of the two clinical programs had a total of 704 episodes of care in which their withdrawal was treated with methadone (Fig. 4). An episode of care refers to receipt of an initial dose of methadone with or without return for subsequent dosing for up to 72 h and referral to ongoing treatment (most commonly at an OTP). In 63 of these episodes, the patient was already connected with an OTP, and so did not require linkage to a new OTP. In the remaining 641 episodes of methadone treatment, 566 patients were linked to ongoing methadone treatment at an OTP within 72 h (566/641 = 88%), while 24 were admitted for management of withdrawal, hospitalized for other reasons, or elected to transition to buprenorphine treatment. A care plan was either not secured or not clearly documented in 51/641 (8%) of episodes, mostly due to the patient not returning to complete referral (26/51), being banned from local OTPs or having other behavioral barriers (e.g., discharged for urinating on floor) (5/51), or detox bed unavailable (4/51). In 16/51 documentation did not clearly indicate the plan of care [22, 23].

Among patients who received clinical services at the RH, 1047 (60.8%) had co-occurring mental health (MH) diagnoses noted in their electronic health record. Non-SUD MH diagnoses recorded in the preceding 2 years include bipolar disorder, schizophrenia and other psychotic disorders, homicidal/suicidal attempt/ideation, personality disorders, traumatic brain injury, conduct disorder, eating disorder, generalized anxiety disorder, panic disorder, post-traumatic stress disorder, major depressive disorder, obsessive–compulsive disorder, and oppositional defiant disorder.

### Clinical care for housing residents

Housing residents were not obliged to utilize the clinical services located in the RH, but many did. Utilization of clinical services could have occurred prior to a person becoming a resident. Of the 100 individuals who resided in RH during the study period, fifty-nine residents (59%) had a total of 425 visits (mean 7.2 visits/resident) in the clinical units. This utilization was concentrated in the first 3 months of residents’ stay in the RH housing. Twenty-six housing residents (26%) were started on methadone for opioid withdrawal management and referred and accepted at an OTP. Overall, 49 residents (49%) engaged with some form of SUD treatment at the RH, at BMC, or in the surrounding community, including medication treatment, counseling, recovery coaching, or residential treatment.

RH housing residents also presented to clinical services with severe acute and chronic health problems. Staff provided residents with emotional and practical support and encouragement to seek a higher level of medical care and to tolerate hospitalization when needed, including in the following cases:

- Endocarditis, acute pulmonary embolus, severe anemia; requiring month-long hospitalization
- Complicated pregnancy and childbirth; neonatal abstinence syndrome
- Endocarditis with triple valve replacement, septic emboli to multiple organs including brain and intervertebral discs, psoas abscess
- Breast mass requiring biopsy
- Surgery for massive, disabling hernia
- Psychiatric hospitalization for psychotic symptoms and suicidality

## Discussion

Our low-threshold transitional housing unit co-located with addiction subspecialty services supported people experiencing homelessness in exiting tent encampments, remaining sheltered, and accessing evidence-based SUD care. The RH housing model, which takes a truly harm-reduction focused approach and allows residents to remain housed even if they continue to use substances is rare in the United States [25]. Many housing programs (both transitional and permanent) for people exiting homelessness remain contingent upon abstinence or treatment engagement. Federal Housing and Urban Development (HUD) [26] policy does not allow federal dollars to be used to house people who have active SUD, and programs that do not rely on federal funding often limit services for people who have active SUD due to fear, moral compunctions, worry about promoting drug use, unfamiliarity, staff resistance, or simply the operational challenges involved [27]. Here, through a collaboration between City, state, academic medical center partners, and collaboration with community-based organizations, we demonstrate that a low-threshold housing approach, co-located with SUD clinical care, supports transitional housing for former encampment residents with extremely high rates of SUD and secured sucessful transition to long term housing for 25% of residents in the first 12 months of the program. Results have broad implication for cities across the country which, like Boston, face interconnected crises of homelessness, SUD, and mental illness.

Several specific aspects of RH program structure likely contributed to housing retention and engagement. Prior work [28] has identified the inability for couples to be housed together as a key factor that perpetuates encampments, since couples are typically required to separate in order to enter programs and/or housing. Many RH residents were members of couples, and they reported anecdotally that being able to stay together in housing made a crucial difference in their decision to enter and remain in the program, since they were not willing to separate from their partner. While RH rules prohibit substance use in the building, instances of substance use were responded to with a clinical and harm reduction-informed approach, including reorientation to RH policies, linkage to SUD treatment and withrawal management care for interested residents, and harm reduction service provision without discharge from housing. Amnesty lockers and policies that allowed housing residents to leave and reenter at any time facilitated residents’ ability to remain in housing during periods of active fentanyl and other substance use. Although treatment programming was available to residents, participation in groups was not required, thereby lowering the barrier to housing for individuals who struggle to meet rigid residential SUD treatment program requirements. Furthermore, on-site SUD clinical services capitalized on moments of readines for treatment entry and supported residents in progress towards health goals. Geographic proximity to OTPs, other medical care, and social services reduced transportation-related barriers. Remarkably, not a single RH housing resident chose to leave and return to living unhoused, defying the popular narrative that people experiencing homelessness “don’t really want to be housed.”

The presence of on-site walk-in clinical services, including a walk-in urgent care bridge clinic focused on low-barrier initiation of medications for SUDs, and a 24/7 short-stay stabilization unit designed to address urgent SUD-related complications, is a unique component of the RH model not available in most low-income housing programs [29]. Although SUD treatment and engagement with clinical services was not required for RH residents, the majority of residents engaged with some form of SUD treatment on site, including a substantial proportion who initiated methadone treatment via opioid withdrawal treatment under the federal “72-h rule.” That methadone withdrawal management was immediately available—an elevator ride away—and supported by the intensive case management needed to link to an OTP for ongoing care—facilitated rapid methadone treatment entry for residents facing well-described barriers to starting this medication for opioid use disorder (MOUD). The model of low-barrier MOUD initiation and on-site 24/7 stabilization services has also helped the vast majority of residents remain safe and alive in spite of extremely high rates of early death from overdoses and other causes that have been documented in people who live unsheltered [30].

Rapidly implementing and maintaining the RH model has involved major challenges. In the housing unit, substantial resources are required to reduce overdose deaths in this very high-risk patient population, both in terms of staffing and operational intensity. In a fentanyl-penetrated drug supply, even very frequent 24/7 room safety checks for each of 60 housing residents, promotion of virtual overdose prevention resources, and robust overdose prevention education and harm reduction service delivery were inadequate to prevent two overdose deaths during the first 12 months of the program. While federal and MA state regulations continue to prohibit supervised consumption, the optimal cadence and approach to wellness checks in low-threhsold housing units remains uncertain; but it is clear that regulatory reform is urgently needed to allow the implementation of evidence-based overdose prevention strategies that are adequate for the realities of our contaminated drug supply [31].

Similarly, the medical acuity of housing residents, who have experienced decompensated cirrhosis, HIV, psychiatric emergencies, complex wounds, infective endocarditis, heart failure, and numerous other acute complications of substance use, in addition to uncontrolled chronic medical conditions, challenged the limits of available services and required close collaboration between housing case managers, social workers, primary care providers, subspecialists, and, when residents chose to access on-site clinical services, RH clinical providers. In several circumstances, residents who met criteria for skilled nursing facilities or other post-acute rehabilitation preferred to return to the RH and demonstrated the capacity to make that medical decision. To further support the needs of housing residents with complex medical and psychiatric conditions, visiting nurse referrals were made and home visits from a Boston Healthcare for the Homeless outreach medical team were instituted. Additionally, community partners from local social service agencies were welcomed to see housing resident clients in the building, with the housing resident’s permission.

Maintaining the safety of RH staff, housing residents, and patients while caring for people with active substance use (including high rates of stimulant use), untreated serious mental illness, and trauma in a neighborhood where structural factors have concentrated the trade of illicit substances, human trafficking, and violence has required very close collaboration of clinical, operations, and security colleagues. RH protocols include allowing only residents and staff to enter the housing unit, metal detector screening for weapons at building entry, and staff utilizing de-escalation techniques to support residents and patients experiencing behavioral health emergencies. For patients in the clinical units, rapid access to medications including benzodiazepines and antipsychotics are available to address psychosis, agitation, and other emergencies. In spite of these precautions, we have experienced several safety events and continue to partner closely across sectors to support safety in the building and surrounding area. As noted above, over the course of the 1st year, 8 housing guests were administratively discharged from the RH prior to obtaining permanent supportive housing. For the most part, these discharges were due to violence against other guests or RH staff. Better access to mental health care could perhaps have averted some of these discharges.

Finally, the RH model involves high operational cost and limited reimbursement for clinical services, leading to challenges for sustainability of the model. Although the Commonwealth of Massachusetts pays for addiction treatment services to manage withdrawal and provide rehabilitation, there is no established daily payment rate for patients such as these who require acute stabilization and linkage to treatment. This makes it difficult to sustain this clinical model financially, in spite of its obvious clinical benefits. Like many programs designed to support people experiencing homelessness and those with SUD, the RH has also elicited intense neighborhood opposition to the housing and clinical programs.

### Study limitations

This study is descriptive, reporting on the feasibility and acceptability of co-located low-barrier housing and clinical stabilization services for patients experiencing homelessness. It does not utilize a control group or compare outcomes to other models of transitional housing, and as such cannot draw causal conclusions between the Roundhouse model and the reported outcomes.

## Conclusions

The combination of low-barrier harm-reduction-focused housing for singles and couples, with clinical stabilization care for substance-related complications, is effective in allowing people who have been living in tent encampments to exit homelessness and achieve increased stabilization in a dignified and relatively safe way.

## Data Availability

The datasets generated and analyzed during the current study are not publicly available due low sample size and privacy of vulnerable individuals but are available from the corresponding author on reasonable request.
